# Comparative analysis of the World Health Organization Reporting System for Head and Neck Cytopathology and the Milan System for Reporting Salivary Gland Cytopathology

**DOI:** 10.1002/cncy.70041

**Published:** 2025-08-25

**Authors:** Adam Kowalewski, Jędrzej Borowczak, Olivier Choussy, Maria Lesnik, Nathalie Badois, Jerzy Klijanienko

**Affiliations:** ^1^ Department of Pathology and Theranostics Institut Curie PSL University Paris France; ^2^ Department of Tumor Pathology Oncology Centre Prof. Franciszek Łukaszczyk Memorial Hospital Bydgoszcz Poland; ^3^ Faculty of Medicine Bydgoszcz University of Science and Technology Bydgoszcz Poland; ^4^ Clinical Department of Oncology Oncology Centre Prof. Franciszek Łukaszczyk Memorial Hospital Bydgoszcz Poland; ^5^ Department of Head and Neck Surgery Institut Curie PSL University Paris France

**Keywords:** cytopathology reporting system, fine‐needle aspiration (FNA), head and neck, Milan System for Reporting Salivary Gland Cytopathology (MSRSGC), risk of malignancy (ROM), salivary gland, World Health Organization (WHO)

## Abstract

**Background:**

A comparative analysis of the International Academy of Cytology–International Agency for Research on Cancer–World Health Organization Reporting System for Head and Neck Cytopathology (WHO) and the Milan System for Reporting Salivary Gland Cytopathology (MSRSGC) was performed.

**Methods:**

A total of 2218 salivary gland fine‐needle aspiration samples collected at the Institut Curie, Paris (1954–2022) were evaluated, with 1356 having histological follow‐up. Samples were classified according to the MSRSGC (nondiagnostic [ND], nonneoplastic [NN], atypia of undetermined significance [AUS], benign neoplasm [BN], salivary gland neoplasm of uncertain malignant potential [SUMP], suspicious for malignancy [SM], and malignant [M]) and the WHO system (insufficient/inadequate/nondiagnostic, benign, atypical, neoplasm of uncertain malignant potential [NUMP], suspicious for malignancy [SM], and malignant [M]). The risk of malignancy (ROM) was calculated for each category, and diagnostic performance metrics were assessed.

**Results:**

In the MSRSGC, the ROM was ND, 50% (*n* = 2); NN, 16.8% (*n* = 149); AUS (no cases); BN, 4.3% (*n* = 514); SUMP, 50% (*n* = 2); SM, 56.1% (*n* = 66); and M, 98.2% (*n* = 623). In the WHO system, the ROM was insufficient/inadequate/nondiagnostic, 50% (*n* = 2); benign, 7.1% (*n* = 663); atypical (no cases); NUMP, 50% (*n* = 2); SM, 56.1% (*n* = 66); and M, 98.2% (*n* = 623). The WHO’s “benign” category, which combines NN and BN, balanced the NN’s higher ROM (16.8%) and BN’s lower ROM (4.3%) into 7.1%. Excluding the ND and SUMP/NUMP categories, both systems demonstrated high diagnostic performance: sensitivity, 93.3%; specificity, 93.9%; positive predictive value, 94.2%; and negative predictive value, 92.9%.

**Conclusions:**

Both systems effectively identify malignancy. The WHO system’s merger of NN and BN into the benign category streamlines reporting and reduces variability, although it may mask clinically significant differences between nonneoplastic and benign neoplastic lesions.

## Abstract 2

A comparative analysis of the International Academy of Cytology–International Agency for Research on Cancer–World Health Organization Reporting System for Head and Neck Cytopathology (WHO) and the Milan System for Reporting Salivary Gland Cytopathology was performed. Both systems effectively identify malignancy. The WHO system’s merger of nonneoplastic and benign neoplastic into the benign category streamlines reporting and reduces variability, although it may mask clinically significant differences between nonneoplastic and benign neoplastic lesions.

## INTRODUCTION

Salivary gland lesions present a complex diagnostic challenge as a result of their diverse histological profiles and the frequent overlap in clinical features between benign and malignant conditions. Fine‐needle aspiration (FNA) has emerged as a vital, minimally invasive technique for the preoperative evaluation of these lesions, which enables clinicians to make informed decisions regarding patient management and treatment strategies.

In 2018, the Milan System for Reporting Salivary Gland Cytopathology (MSRSGC) was established to standardize the classification and reporting of salivary gland FNA samples.^1^, ^2^ This evidence‐based framework organizes FNA findings into seven categories: nondiagnostic (ND), nonneoplastic (NN), atypia of undetermined significance (AUS), benign neoplasm (BN), salivary gland neoplasm of uncertain malignant potential (SUMP), suspicious for malignancy (SM), and malignant (M). Each category is linked to a specific risk of malignancy (ROM), which enhances communication between pathologists and clinicians and supports the identification of benign conditions, and thereby reduces unnecessary surgical interventions and guides subsequent therapeutic approaches.^3^

However, despite its widespread acceptance and benefits, the MSRSGC faces limitations that can undermine diagnostic precision. Factors such as suboptimal sampling techniques, insufficient tissue yield, procedural artifacts, and the intrinsic heterogeneity of salivary gland tumors contribute to diagnostic uncertainty. To mitigate these issues, the system incorporates inconclusive categories—ND, AUS, and SUMP—which, although addressing some diagnostic challenges, result in inconclusive outcomes in up to 30% of cases.^4^ These indeterminate diagnoses frequently necessitate additional procedures, such as repeat FNA or surgical excision with histological follow‐up, which elevate patient anxiety, health care costs, and the cumulative risk of procedural complications.^5^

In an effort to unify the field of head and neck cytopathology, the International Academy of Cytology, International Agency for Research on Cancer, and World Health Organization (WHO) introduced the WHO Reporting System for Head and Neck Cytopathology during the United States and Canadian Academy of Pathology annual meeting in 2025. Although this system has not yet been officially published, it represents a major initiative to integrate cytological classification across all head and neck sites. This reporting framework is designed to complement site‐specific systems, such as the MSRSGC for salivary gland cytology, and is part of a broader WHO series integrating published evidence and cytopathology practice, linked to the WHO Classification of Tumours series. It provides a standardized, hierarchical framework of diagnostic categories, each associated with management recommendations to improve clinical communication and patient care globally. The WHO system seeks to enhance diagnostic clarity by refining category structures, including the consolidation of the MSRSGC’s NN and BN categories into a unified “benign” category, alongside other designations such as insufficient/inadequate/nondiagnostic, atypical, neoplasm of uncertain malignant potential (NUMP), suspicious for malignancy (SM), and malignant (M). This restructuring aims to streamline reporting and reduce interpretive variability, although its implications for clinical practice require further exploration.

The present study aims to compare the MSRSGC and the WHO reporting system by analyzing a large cohort of salivary gland FNA samples with histological follow‐up. By assessing the ROM across categories and evaluating diagnostic performance metrics, we seek to determine whether the WHO system’s modifications provide superior clinical utility and diagnostic accuracy compared to the established Milan system, and potentially offer a more effective framework for managing salivary gland lesions.

## MATERIALS AND METHODS

### Sample acquisition and examination

A retrospective review was conducted on 2218 FNA samples of salivary glands and 1356 corresponding histological specimens, collected at the Institut Curie in Paris between 1954 and 2022. All FNA procedures were performed by surgical pathologists. Because of the broad time frame of the study, comprehensive details on sampling techniques for each case were not uniformly documented. Before 1995, FNAs were generally performed with palpation guidance.^6^, ^7^ Since 1995, ultrasound guidance has been used for most pediatric cases, typically under local skin anesthesia.^8^

Upon inclusion, all cytological specimens were reassessed and categorized per the second edition of the MSRSGC guidelines, which include the classifications ND, NN, AUS, BN, SUMP, SM, and M.^9^ Additionally, specimens were evaluated according to the fifth edition of the WHO Classification of Head and Neck Tumours, which encompasses categories such as insufficient/inadequate/nondiagnostic, benign, atypical, NUMP, SM, and M.^10^ All matched histological sections were reviewed, with tumors reclassified according to WHO fifth edition tumor terminology, regardless of the original diagnosis.^10^, ^11^

### Sample staining

Cytological samples were stained predominantly with the classic May–Grünwald–Giemsa method, supplemented occasionally by Papanicolaou staining. In recent years, the Diff‐Quik method has been adopted, particularly for FNA quality assurance (rapid on‐site evaluation). Although routine cytological evaluation was based on conventional stains, cell blocks were prepared in selected newer cases, which allowed for immunocytochemistry when diagnostically indicated. Nevertheless, the assignment of Milan categories was strictly based on cytomorphological criteria according to the second edition of the MSRSGC guidelines.^9^

Histological assessments used slides from matching core needle biopsies, surgical biopsies (in earlier cases), or surgical specimens, stained with hematoxylin, eosin, and saffron. Since the late 1990s, molecular techniques and immunohistochemical analyses became available and were applied when needed to refine the final diagnosis. Where available, these findings contributed to the final cytological or histological diagnosis.

### Inclusion and exclusion criteria

The analysis included all samples diagnosed cytologically or histologically as salivary gland tumors. Cases were included regardless of histological follow‐up for descriptive purposes and categorization. However, for the statistical evaluation of diagnostic performance, only cases with available histopathological follow‐up were analyzed. Accordingly, the main exclusion criterion was the absence of histopathological follow‐up for malignant tumors or those with low or uncertain malignant potential. Benign or nonneoplastic FNA diagnoses lacking histological correlation were included if supported by reliable clinical follow‐up and radiological evaluation.

Cytological diagnoses were obtained from the original clinical reports. Relevant clinicopathological data, including age, sex, tumor location, and final histopathological diagnosis, were extracted from hospital records.

### Statistical analysis

Statistical analysis was conducted with Statistica, version 13.3 and Microsoft Excel 2021. For calculating FNA predictive values, the SM and M categories were designated as “positive” cytopathological tests for malignancy, whereas NN and BN were considered “negative.” No AUS samples were present in the cohort. ND or SUMP samples were excluded from these calculations because of their indeterminate malignant potential. Predictive values are reported with their corresponding 95% confidence intervals.

## RESULTS

### General findings

A total of 2218 FNA samples were analyzed that were collected between 1954 and 2015. Of these, 1727 cases (77.9%) were collected from the parotid gland, 271 cases (12.2%) from the submandibular gland, and 126 cases (5.7%) from the minor salivary glands, and 78 cases (3.5%) had an unspecified site of origin (Table 1). The median patient age was 58 years, with ages ranging from 3 months to 98 years.

**TABLE 1:** Distribution of fine‐needle aspiration samples by salivary gland location.

Site	Cases (*N* = 2218), No. (%)
Parotid gland	1727 (77.9)
Submandibular gland	271 (12.2)
Sublingual gland	4 (0.2)
Submental gland	3 (0.1)
Subdigastric gland	9 (0.4)
Minor salivary glands	126 (5.7)
Not specified	78 (3.5)

Initial classification was performed via the MSRSGC (Table 2). Conclusive cytological diagnoses were achieved in 99.7% of cases, with three cases classified as ND, 554 cases as NN, 760 cases as BN, three cases as SUMP, 76 cases as SM, and 823 cases as M. No samples were classified as AUS. Cytopathological examination alone was sufficient for a final diagnosis in 862 cases (38.9%), including 403 NN cases (46.8%), 253 BN cases (29.4%), and 206 M cases (23.9%).

**TABLE 2:** Fine‐needle aspiration classification by Milan system categories with histopathological follow‐up.

Category	ND	NN	AUS	BN	SUMP	SM	M	Total
Cases, No. (%)	3 (0.1)	553 (24.9)	0	760 (34.3)	3 (0.1)	76 (3.4)	823 (37)	2218
Cases with histological follow‐up, No. (%)	2 (0.1)	149 (11)	0	514 (37.9)	2 (0.1)	66 (4.9)	623 (45.9)	1356 (61.1)
Benign: nonneoplastic, No. (%)	0	91 (61.1)	0	10 (1.9)	0	7 (10.6)	2 (0.3)	110 (8.1)
Benign: neoplastic, No. (%)	1 (50)	33 (22.2)	0	482 (93.8)	1 (50)	22 (33.3)	9 (1.4)	548 (40.4)
Malignant, No. (%)	1 (50)	25 (16.8)	0	22 (4.3)	1 (50)	37 (56.1)	612 (98.2)	698 (51.5)
Risk of neoplasia, %	100	38.9	—	98.1	100	89.4	99.7	91.9
Risk of malignancy, %	50	16.8	—	4.3	50	56.1	98.2	51.5

Abbreviations: AUS, atypia of undetermined significance; BN, benign neoplasm; M, malignant; ND, nondiagnostic; NN, nonneoplastic; SM, suspicious for malignancy; SUMP, salivary gland neoplasm of uncertain malignant potential.

Histological follow‐up was available for 1356 cases (61.1%), which confirmed 110 NN, 548 BN, and 698 M diagnoses. Among the ND category, one of two samples with follow‐up was malignant (50%). In the NN category, 25 of 149 samples with follow‐up were malignant (16.8%). The BN category showed 22 M cases (4.3%) among 514 samples with follow‐up, whereas one of two SUMP cases was malignant (50%). In the SM category, 37 of 66 samples were malignant (56.1%), and in the M category, 11 of 623 samples were nonmalignant (1.8%).

Samples were then reclassified via the WHO Reporting System for Head and Neck Cytopathology (Table 3), which resulted in three cases categorized as insufficient/inadequate/nondiagnostic, 1313 cases as benign, three cases as NUMP, 76 cases as SM, and 823 cases as M. A histological correlate was not available in 862 cases (38.9%), for which the cytological diagnosis was accepted as final. The results comprised 656 benign cases (76.1%) and 206 M cases (23.9%). Among the 1356 cases with histological follow‐up, 656 were benign (48.5%) and 698 were malignant (51.5%). In the insufficient/inadequate/nondiagnostic category, one of two cases was malignant (50%). In the benign category, 47 of 663 samples with follow‐up were malignant (7.1%), whereas one of two NUMP cases was malignant (50%) and 37 of 66 SM cases were malignant (56.1%). In the M category, 11 of 623 samples were nonmalignant (1.8%).

**TABLE 3:** Fine‐needle aspiration classification by WHO reporting system categories with histopathological follow‐up.

Category	Insufficient/inadequate/nondiagnostic	Benign	AUS	NUMP	SM	M	Total
Cases, No. (%)	3 (0.1)	1313 (59.2)	0	3 (0.1)	76 (3.4)	823 (37)	2218
Cases with histological follow‐up, No. (%)	2 (0.1)	663 (50.5)	0	2 (66.7)	66 (86.8)	623 (75.7)	1356 (61.1)
Benign (inflammatory or neoplastic), No. (%)	1 (50)	616 (92.9)	0	1 (50)	29 (43.9)	11 (1.8)	658 (48.5)
Malignant, No. (%)	1 (50)	47 (7.1)	0	1 (50)	37 (56.1)	612 (98.2)	698 (51.5)
Risk of malignancy, %	50	7.1	—	50	56.1	98.2	51.5

Abbreviations: AUS, atypia of undetermined significance; M, malignant; NUMP, neoplasm of uncertain malignant potential; SM, suspicious for malignancy.

M cases included 82 adenoid cystic carcinomas, 70 non‐Hodgkin lymphomas, 67 mucoepidermoid carcinomas, 43 carcinoma ex pleomorphic adenomas, 31 acinic cell carcinomas, 276 metastases, and 149 other cancers. Among metastases, squamous cell carcinoma was the most common (40.2%; 111 of 276), followed by melanoma (26.8%; 74 of 276), breast cancer (6.5%; 18 of 276), and retinoblastoma (3.6%; 10 of 276). A full list of malignancies is provided in Table S1.

For conclusive MSRSGC categories (II, IVa, V, and VI) with follow‐up, the sensitivity, specificity, positive predictive value (PPV), and negative predictive value (NPV) for diagnosing malignancy were 93.3%, 93.9%, 94.2%, and 92.9%, respectively (Table 4). False negatives were most frequently mucoepidermoid carcinoma (28.5%; 14 of 49), followed by carcinoma ex pleomorphic adenoma (8.2%; four of 49), acinic cell carcinoma (6.1%; three of 49), adenoid cystic carcinoma (6.1%; three of 49), and non‐Hodgkin lymphoma (6.1%; three of 49) (Table 5). False positives were dominated by pleomorphic adenoma (45%; 18 of 40), followed by Warthin tumor (7.5%; three of 40), basal cell adenoma (5%; two of 40), inflammation (5%; two of 40), immunoglobulin 4 (IgG4)–related disease (5%; two of 40), nodular fasciitis (5%; two of 40), and tuberculosis (5%; two of 40) (Table 6). Detailed cytological and histological diagnoses for false negatives and false positives are available in Tables S2 and S3.

**TABLE 4:** Diagnostic performance metrics of FNA for distinguishing malignant from nonmalignant salivary gland lesions.

Predictive values of FNA (SUMP excluded)^a^		Histopathology		Predictive values of FNA (SUMP included)^a^		Histopathology	
		Malignant	Nonmalignant			Malignant	Nonmalignant
Malignant, No.		650	40	Malignant, No.		650	40
Nonmalignant, No.		47	615	Nonmalignant, No.		49	617
Sensitivity, % (95% CI)	93.3	(91.1–95)		Sensitivity, % (95% CI)	93	(90.8–94.8)	
Specificity, % (95% CI)	93.9	(91.8–95.6)		Specificity, % (95% CI)	93.9	(91.8–95.6)	
PPV, % (95% CI)	94.2	(92.3–95.6)		PPV, % (95% CI)	94.2	(92.6–95.6)	
NPV, % (95% CI)	92.9	(90.8–94.5)		NPV, % (95% CI)	93.2	(92.6–94.3)	
LR (+) (95% CI)	15.3	(11.3–20.6)		LR (+) (95% CI)	15.3	(11.3–20.6)	
LR (−) (95% CI)	0.07	(0.05–0.09)		LR (−) (95% CI)	0.07	(0.06–0.1)	
Accuracy, % (95% CI)	93.6	(92.1–94.8)		Accuracy, % (95% CI)	93.4	(92–94.7)	
Cohen κ (95% CI)	0.87	(0.85–0.90) σ = 0.01		Cohen κ	0.87	(0.84–0.90) σ = 0.01	

Abbreviations: FNA, fine‐needle aspiration; LR (+), positive likelihood ratio; LR ( ), negative likelihood ratio; NPV, negative predictive value; PPV, positive predictive value; SUMP, salivary gland neoplasm of uncertain malignant potential.

^a^ None of the study cases were classified as atypia of undetermined significance.

**TABLE 5:** False negatives in fine‐needle aspiration diagnosis by frequency.

Type of malignancy	False negatives, No.	Total, No.	False negative ratio, %
Mucoepidermoid carcinoma	14	67	20.9
Carcinoma ex pleomorphic adenoma	10	35	28.6
Carcinoma ex pleomorphic adenoma, NOS	4	18	22.2
Mucoepidermoid carcinoma, ex PA^a^	2	11	18.2
Myoepithelial carcinoma, ex PA	2	2	100
Clear cell carcinoma, ex PA	1	3	33.3
Squamous cell carcinoma, ex PA	1	1	100
Squamous cell carcinoma	4	111	3.6
Acinic cell carcinoma	3	31	9.7
Adenoid cystic carcinoma	3	82	3.7
Non‐Hodgkin lymphoma	3	70	4.3
Carcinoma, NOS	2	23	8.7
Low‐grade papillary adenocarcinoma	2	17	11.8
Basaloid squamous cell carcinoma	1	5	20
Malignant lymphoma, ex Warthin	1	1	100
Melanoma	1	72	1.4
Leiomyosarcoma	1	2	50
Osteosarcoma	1	2	50
Myoepithelial carcinoma	1	6	16.7
Papillary thyroid carcinoma	1	3	33.3
Polymorphous adenocarcinoma (coexisting with PA)	1	1	100

Abbreviations: NOS, not otherwise specified; PA, pleomorphic adenoma.

^a^ The concept of “mucoepidermoid carcinoma ex pleomorphic adenoma” is not well accepted because it is typically *MAML2*‐driven and not derived from *PLAG1*‐ or *HMGA2*‐positive pleomorphic adenomas.

**TABLE 6:** False positives in fine‐needle aspiration diagnosis by frequency.

Type of lesion	False positives, No.	Total, No.	False positive ratio, %
Pleomorphic adenoma	18	389	4.6
Warthin tumor	3	84	3.6
Basal cell adenoma	2	24	8.3
Inflammation	2	22	9.1
IgG4‐related disease	2	12	16.7
Nodular fasciitis	2	2	100
Tuberculosis	2	11	18.2
Adamantinoma	1	2	50
Benign lymphoepithelial lesion	1	5	20
Epidermal cyst	1	24	4.2
Fibromatosis	1	3	33.3
Inverted ductal papilloma	1	1	100
Pilomatricoma	1	5	20
Mucocele	1	1	100
Myoepithelioma	1	4	25
Schwannoma	1	3	33.3

Abbreviation: IgG4, immunoglobulin 4.

### Detailed results per MSRSGC category

#### Category I: ND

Only three samples were classified as ND, with two undergoing histopathological follow‐up. One was identified as acinic cell carcinoma, and the other showed inflammatory changes. The overall malignancy rate was 33.3% (one of three), which increased to 50% (one of two) in cases with follow‐up.

#### Category II: NN

**FIGURE 1:**
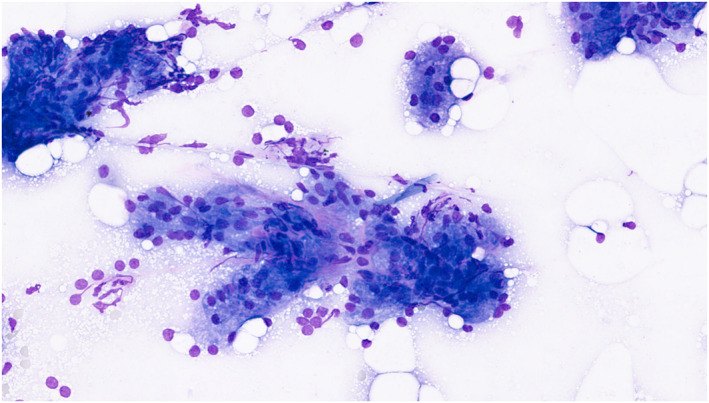
Milan II nonneoplastic and World Health Organization benign categories. Nonneoplastic sialadenosis. Acinic cells and naked nuclei should be differentiated from well‐differentiated acinic cell carcinoma. May–Grünwald–Giemsa.

**FIGURE 2:**
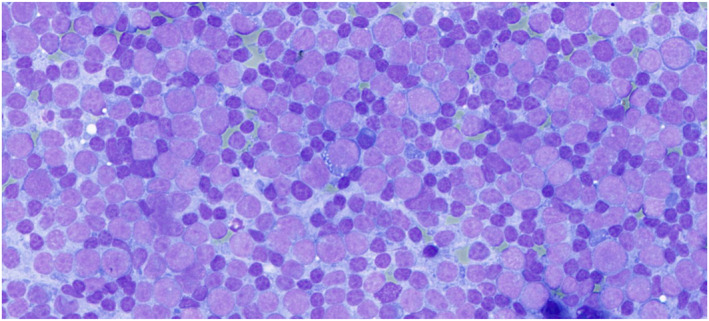
Milan II nonneoplastic and World Health Organization benign categories. Nonneoplastic parotid inflammatory lymph node, which may simulate clinically and radiologically a salivary tumor. May–Grünwald–Giemsa.

A total of 553 FNA samples were diagnosed as NN, including hyperplasia (29.5%; 163 of 553), inflammatory changes (25.5%; 141 of 553), epithelial cysts (15.9%; 88 of 553), sarcoidosis (6.1%; 34 of 553), and IgG4‐related disease (3.6%; 20 of 553) (Figures 1 and 2). Of these, 149 (26.9%) had histopathological follow‐up, which revealed 25 M cases (16.8%), including 11 mucoepidermoid carcinomas, three non‐Hodgkin lymphomas, four squamous cell carcinomas, one leiomyosarcoma, one mucoepidermoid carcinoma ex pleomorphic adenoma, one malignant lymphoma ex Warthin tumor, and one melanoma, plus 33 benign tumors (Table S2). The overall ROM was 4.9% (27 of 553) and the risk of neoplasia was 12.3% (68 of 553), compared to 16.8% and 38.9% for samples with follow‐up, respectively.

#### Category III: AUS

**FIGURE 3:**
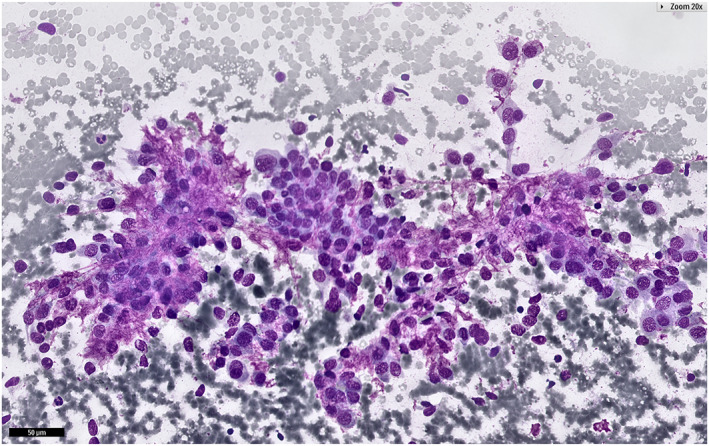
Milan III, atypia of undetermined significance and World Health Organization atypical categories. Myoepithelioma with atypical cells, which should be differentiated from carcinoma ex pleomorphic adenoma. May–Grünwald–Giemsa.

No samples were classified as AUS (Figure 3). Cases lacking malignancy features were categorized as NN or BN, unclear diagnoses as SM, and those with malignancy features as M.

#### Category IVa: BN

**FIGURE 4:**
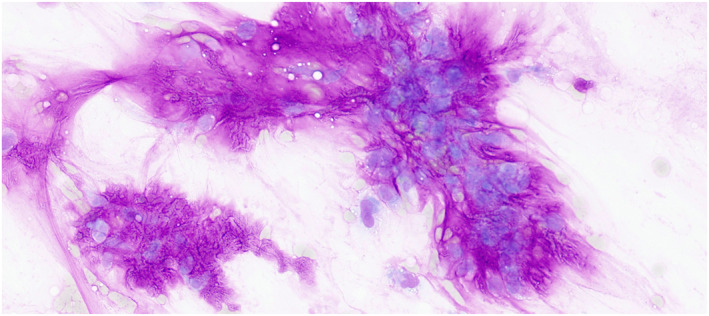
Milan IVa BN and World Health Organization benign categories. Pleomorphic adenoma composed from isolated myoepithelial cells and a chondromyxoid background. May–Grünwald–Giemsa. This illustrative case is from an external cohort, and was not included in the main study data set.

A total of 760 cases were diagnosed as BN, primarily pleomorphic adenoma (64.5%; 490 of 760), Warthin tumor (20.3%; 154 of 760), basal cell adenoma (4.2%; 32 of 760), and lipoma (1.6%; 12 of 760) (Figure 4). Of these, 514 had surgical follow‐up. Among 490 cases initially identified as pleomorphic adenoma, 360 (73.5%) underwent histopathological examination, which identified 18 malignancies (11 carcinoma ex pleomorphic adenomas, two adenoid cystic carcinomas, two acinic cell carcinomas, two low‐grade papillary adenocarcinomas, and one basaloid cell carcinoma) and 10 NN cases (Table S2). The overall ROM was 3.2% and the risk of neoplasia was 98.4%, compared to 4.8% and 98.1% for samples with follow‐up, respectively.

#### Category IVb: SUMP

Three samples were classified as SUMP on the basis of cytological features consistent with neoplasia but insufficient to confidently distinguish between benign and malignant entities. The initial cytological diagnoses were basal cell adenoma, fibroma, and fusiform cells. Two of these cases (66.7%) were resected: the fusiform cell sample was myoepithelial carcinoma, and the fibroma was pericytoma. The overall ROM in this category was 33.3% and the risk of neoplasia was 100%, compared to 50% and 100%, respectively, in the subset with follow‐up.

#### Category V: SM

**FIGURE 5:**
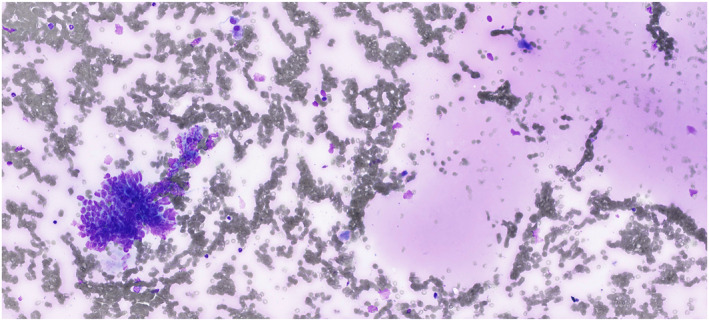
Milan V suspicious for malignancy and World Health Organization suspicious categories. Pleomorphic adenoma and a mucoid background. It should be differentiated from mucoepidermoid carcinoma ex pleomorphic adenoma. May–Grünwald–Giemsa.

Seventy‐six cases were SM; all cases met the strict diagnostic criteria for SM on the basis of cytomorphological features alone. The most common cytological interpretations included pleomorphic adenoma with atypia (21.1%; 16 of 76), mucoepidermoid carcinoma (10.5%; eight of 76), carcinoma ex pleomorphic adenoma (6.6%; five of 76), acinic cell carcinoma (6.6%; five of 76), non–otherwise specified carcinoma (5.3%; four of 76), melanoma (3.9%; three of 76), and tuberculosis (3.9%; three of 76) (Figure 5). The latter cases exhibited marked atypia, necrosis, or architectural disorder, which warranted their classification as SM. Of these, 66 (86.8%) had histopathological follow‐up, which revealed 29 false negatives (seven NN; 22 BN) and 37 M cases (56.1%), including myoepithelial carcinoma (18.9%; seven of 37), acinic cell carcinoma (13.5%; five of 37), and carcinoma ex pleomorphic adenoma (10.8%; four of 37). Of the 16 cases initially identified as pleomorphic adenoma with atypia, three had no histopathological follow‐up but auxiliary studies confirmed malignancy in two of them. All of the remaining 13 pleomorphic adenomas had surgical follow‐up that confirmed their benign nature (Table S3). Other common false positives included two Warthin tumors (6.9%) and two tuberculosis cases (6.9%). One sample, collected from a 4‐year‐old patient, was classified as SM on the basis of high cellularity and nuclear atypia on FNA, and an atypical conjunctival lesion was diagnosed. Histological follow‐up revealed nodular fasciitis. The overall ROM was 53.9% and the risk of neoplasia was 84.2%, compared to 56.1% and 89.4% in samples with follow‐up, respectively.

#### Category VI: M

**FIGURE 6:**
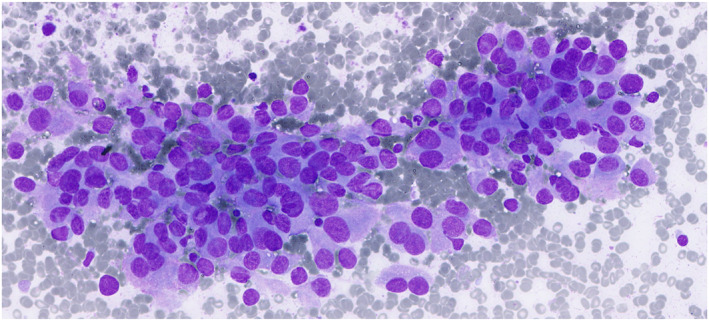
Milan VI malignant and World Health Organization malignant categories. Malignancy is evident in smears from salivary duct carcinoma. May–Grünwald–Giemsa.

A total of 823 cases were diagnosed as M, including metastatic cancers (45.8%; 377 of 823), adenoid cystic carcinomas (10.7%; 88 of 823), mucoepidermoid carcinomas (6.3%; 52 of 823), acinic cell carcinomas (3.4%; 28 of 823), and carcinoma ex pleomorphic adenomas (2.8%; 23 of 823) (Figure 6). Of these, 623 had surgical follow‐up, which confirmed metastases (39.5%; 276 of 698), adenoid cystic carcinomas (11.7%; 82 of 698), and non‐Hodgkin lymphomas (10%; 70 of 698) as the most common. The high proportion of metastatic tumors in our cohort reflects the referral profile of our institution, where many salivary gland FNAs targeted parotid region lymph nodes or adjacent soft tissue masses in patients with suspected secondary tumors. There were 11 false positives: five pleomorphic adenomas, two cases of IgG4‐related disease, one Warthin tumor, one inverted ductal papilloma, one basal cell adenoma, and one nodular fasciitis (Table S3). Of the 823 cases, 809 were confirmed malignant, which yielded an overall ROM of 98.3%, compared to 98.2% in samples with follow‐up.

## DISCUSSION

At our institution, the MSRSGC applied to FNA demonstrated robust diagnostic performance, with a sensitivity of 93.3% and a specificity of 93.9% for detecting salivary gland malignancies. These values are comparable to or exceed those reported in several recent studies, including Lubin et al. (65.6% sensitivity; 87.4% specificity) and Palacios‐Garcia et al. (99% sensitivity; 55% specificity).^12^, ^13^, ^14^ While Palacios‐Garcia et al. reported higher sensitivity, our cohort demonstrated a more favorable trade‐off between sensitivity and specificity. Notably, Lubin et al. used the same approach as ours for calculating diagnostic performance, with the exclusion of ND cases and the requirement of histological follow‐up. They applied two definitions of “cytologically malignant” (M alone or SM plus M), with the latter matching our criteria. Although Palacios‐Garcia et al. also included SUMP as malignant, this had minimal impact on comparability because our cohort included no AUS cases and only one SUMP case with histological follow‐up. The PPV and NPV were 94.2% and 92.9%, respectively, consistent with ranges reported in the literature (71%–100% PPV; 72%–98% NPV).^4^, ^12^, ^13^, ^15^, ^16^ However, our cohort exhibited a higher malignancy rate—37.1% (823 of 2218) across all FNA samples and 45.9% (698 of 1356) among those with histopathological follow‐up—compared to the 13.3% reported in a meta‐analysis of 7168 samples by Wang et al.^4^ This elevated malignancy prevalence likely contributed to a slightly lower NPV compared to studies with fewer M cases.^4^

The MSRSGC categorizes salivary gland lesions to estimate ROM, with initial values set at 25% (ND), 10% (NN), 20% (AUS), 5% (BN), 35% (SUMP), 60% (SM), and 90% (M).^17^ Updated in 2024 on the basis of more than 200 studies, the system reflects broader data.^9^ In our cohort, the ROM for NN was higher (16.8% vs. 10%–11%) and for SM was lower (56.1% vs. 60%) than the updated Milan benchmarks (Table 7). These variations fall within ranges reported elsewhere, and may stem from the high malignancy rate (51.5%) among samples with histopathological follow‐up, as well as institutional diagnostic preferences favoring definitive categorization. For context, Wang et al. reported an overall ROM of 25.7%, and Rossi et al. noted 21.7%.^4^, ^17^ The scarcity of ND (0.2%; six of 2218) and SUMP (0.2%) cases, and the absence of AUS, limits their comparison to studies reporting up to 30% inconclusive diagnoses. Given the lack of published ROM data for the WHO reporting system, our results offer an early reference point for future validation and comparison.

**TABLE 7:** Comparison of risk of malignancy by Milan category in recent studies.

Study	ND, %	NN, %	AUS, %	BN, %	SUMP, %	SM, %	M, %
Institut Curie cohort	50^a^	16.8	—	4.3	50^a^	56.1	98.2
Milan system 1st edition^16^	25	10	20	5	35	60	90
Milan system 2nd edition^9^	15	11	30	<3	35	83	>98
Wang 2022 meta‐analysis^4^	11.4	10.9	30.5	2.8	37.7	83.8	97.7
Farahani & Baloch 2019 meta‐analysis^18^	17	8	34	4	42	58	91
Jalaly 2020 literature review^19^	16.9	10.5	39.9	2.9	39.4	84.2	97.5
Wei 2017 review^20^	25	10.2	—	3.4	37.5	58.6	91.9

Abbreviations: AUS, atypia of undetermined significance; BN, benign neoplasm; M, malignant; ND, nondiagnostic; NN, nonneoplastic; SM, suspicious for malignancy; SUMP, salivary gland neoplasm of uncertain malignant potential.

^a^ Low number of cases.

The WHO Reporting System for Head and Neck Cytopathology, recently introduced, refines the MSRSGC framework. Its insufficient/inadequate/nondiagnostic category aligns with the MSRSGC’s ND, which aids comparability across classifications and highlights sample adequacy issues. Because false‐positive and ‐negative cases were concordantly categorized under both the MSRSGC and WHO systems, with no reassignments that would alter their diagnostic status, ROM values remained comparable across systems. However, merging NN and BN into a single benign category yielded a ROM of 7.1% in our study (Table 8), which smoothed discrepancies between the NN (16.8%) and BN (4.3%) categories in the MSRSGC.^21^ This consolidation may enhance reporting consistency, although its utility in high‐malignancy cohorts like ours requires further validation, given our elevated baseline ROM (Table 6).

**TABLE 8:** Risk of malignancy comparison between Milan system^21^ and WHO reporting system categories.

MSRSGC	ROM, %	WHO Reporting System for Head and Neck Cytopathology	ROM, %
ND	50^a^	Insufficient/inadequate/nondiagnostic	50^a^
NN	16.8	Benign	7.1
BN	4.3		
AUS	—	AUS	—
SUMP	50	NUMP	50
SM	56.1	SM	56.1
M	98.2	M	96.6

Abbreviations: AUS, atypia of undetermined significance; BN, benign neoplasm; M, malignant; MSRSGC, Milan System for Reporting Salivary Gland Cytopathology; ND, nondiagnostic; NN, nonneoplastic; NUMP, neoplasm of uncertain malignant potential; ROM, risk of malignancy; SM, suspicious for malignancy; SUMP, salivary gland neoplasm of uncertain malignant potential; WHO, World Health Organization.

^a^ Low number of cases.

The ND category, indicative of suboptimal samples, had a ROM of 50% in our study, which reinforces the need for repeat biopsy. Only 0.2% of our samples were ND, likely because of ultrasound guidance and rapid on‐site evaluation, which reduce ND rates.^22^, ^23^ Specimen adequacy remains a subjective judgment by cytopathologists^9^, ^21^ but standardized protocols could further minimize such cases.

**FIGURE 7:**
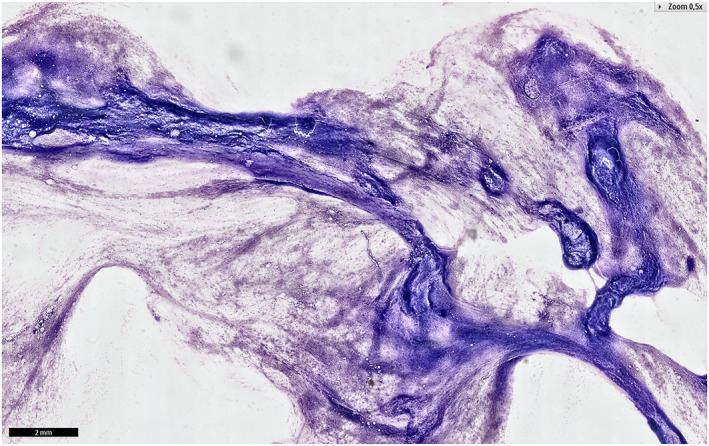
Cytology image of a mucoepidermoid carcinoma initially categorized as nonneoplastic. Smears are composed of mucus, epithelial cells are absent, and there are no malignancy features, which contributed to the diagnostic error.

In the NN category, the ROM was 4.9% across all samples but rose to 16.8% with histopathological follow‐up, which suggests selection bias toward surgically resected cases with higher malignancy suspicion. This may reflect practices at high‐volume oncology centers versus less specialized settings. Mucoepidermoid carcinoma caused 44% (11 of 25) of false negatives in NN, with 11 of 14 missed cases initially labeled NN (Table 5). For instance, Figure 7 illustrates a challenging case of mucoepidermoid carcinoma that was initially misclassified as NN because of the presence of bland epithelial cells and the absence of overt malignancy features, which highlights the difficulty in distinguishing it from benign cysts. Overall, 20.9% (14 of 69) of mucoepidermoid carcinomas were misdiagnosed, which underscores challenges in distinguishing them from benign cysts.^24^, ^25^, ^26^ Non‐Hodgkin lymphoma, the second most common false negative (12%; three of 25), aligns with prior findings.^14^, ^27^

No cases were assigned to the AUS category because none of the cytological specimens fulfilled the criteria for this classification under the MSRSGC. Although the reclassification was conducted independent of the original diagnoses, the available material and institutional diagnostic tendencies may have contributed to bias toward more definitive diagnoses, especially in borderline cases. Therefore, some cases that might have been considered AUS in other settings were categorized as NN, SM, or M, depending on the dominant cytological features. This may have contributed to the lower ROM in the SM category (56.1%) compared to other studies (Table 7).

Similarly, most cases with features suggestive of the SUMP category were reclassified into more specific Milan categories on the basis of the presence of morphological or architectural clues if a more confident diagnosis was feasible. Only a small subset of cases were retained as SUMP because of persistent ambiguity despite review.

**FIGURE 8:**
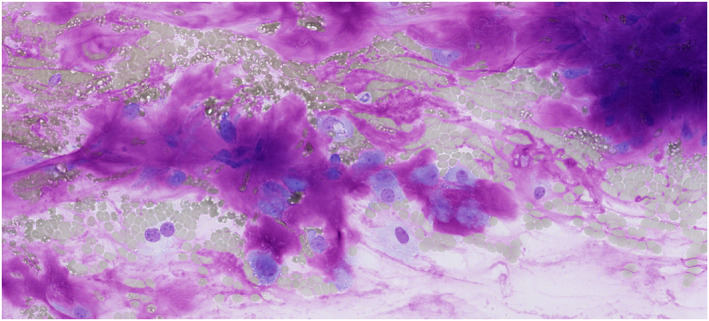
Cytology image of a pleomorphic adenoma displaying heterogeneous features, including prominent myoepithelial cells and atypical nuclear morphology, which illustrates the cytological variability that can lead to misdiagnosis as malignant.

Pleomorphic adenoma, which comprised 64.5% (490 of 760) of BN cases, frequently led to diagnostic errors. Of 360 with follow‐up, 18 (5%) were malignant, including 11 carcinoma ex pleomorphic adenomas (61.1%). Across the cohort, 36 such cancers were identified, with 11 missed initially, which made pleomorphic adenoma the top source of false negatives (Table 5). It also caused 45% (18 of 40) of false positives, with 4.6% (18 of 389) misdiagnosed as M. Its heterogeneity—varying myoepithelial, epithelial, ductal, or nuclear features—complicates cytological assessment.^28^ Figure 8 exemplifies this variability by showing a pleomorphic adenoma with prominent myoepithelial components and atypical nuclear features, which can be mistaken for a malignant neoplasm. Furthermore, the evolving understanding of salivary gland neoplasms is exemplified by entities such as secretory carcinoma, which was likely classified as acinic cell carcinoma in the past because of their histopathological similarities. This underscores the importance of ongoing refinements in classification systems, such as the MSRSGC and the WHO reporting system, to improve diagnostic accuracy and reflect the latest advancements in cytopathology.

In conclusion, both the Milan system and the WHO reporting system effectively identify malignancy in salivary gland FNA samples. The WHO system's unified benign category, which merges NN and BN, simplifies reporting and enhances consistency but may obscure key management differences between these lesions. Although low inconclusive diagnosis rates reflect strong techniques, challenges persist in accurately diagnosing certain tumors, which necessitates further validation of the WHO system and advanced diagnostic tools.

## AUTHOR CONTRIBUTIONS

**Adam Kowalewski:** Conceptualization; writing—original draft; methodology; software; data curation; supervision; and formal analysis. **Jędrzej Borowczak:** Conceptualization; investigation; writing—original draft; software; data curation; supervision; formal analysis; and methodology. **Olivier Choussy:** Conceptualization; investigation; and methodology. **Maria Lesnik:** Conceptualization; investigation; and methodology. **Nathalie Badois:** Conceptualization; investigation; and methodology. **Jerzy Klijanienko:** Conceptualization; writing—original draft; investigation; methodology; validation; data curation; and supervision.

## CONFLICT OF INTEREST STATEMENT

The authors declare no conflicts of interest.

## Supporting information

Table S1

Table S2

Table S3
