# Supplementary material for: Comparative analysis of the World Health Organization Reporting System for Head and Neck Cytopathology and the Milan System for Reporting Salivary Gland Cytopathology
Source: Cancer Cytopathol. 2025 Aug 25;133(9):e70041. doi: 10.1002/cncy.70041 (PMC12377497; doi:10.1002/cncy.70041)
Supplement: Supplementary file 1 — Table S1 [file CNCY-133-0-s003.docx]

**Supplementary Table 1.** Histopathologically confirmed salivary gland malignancies

| **Diagnosis** | **No. of cases (n = 698)** |
| --- | --- |
| Adenoid cystic carcinoma | 82 |
| Non-Hodgkin lymphoma | 70 |
| Mucoepidermoid carcinoma | 67 |
| Carcinoma ex pleomorphic adenoma | 43 |
| Carcinoma ex pleomorphic adenoma, NOS | 18 |
| Mucoepidermoid carcinoma ex pleomorphic adenoma* | 11 |
| Adenoid cystic carcinoma ex pleomorphic adenoma | 4 |
| Clear cell carcinoma ex pleomorphic adenoma | 3 |
| Myoepithelial carcinoma ex pleomorphic adenoma | 2 |
| Salivary duct carcinoma ex pleomorphic adenoma | 2 |
| Acinic cell carcinoma ex pleomorphic adenoma | 2 |
| Squamous cell carcinoma ex pleomorphic adenoma | 1 |
| Acinic cell carcinoma | 31 |
| Salivary duct carcinoma | 28 |
| Carcinoma, NOS | 23 |
| Low-grade papillary adenocarcinoma | 17 |
| Small cell neuroendocrine carcinoma | 7 |
| Myoepithelial carcinoma | 6 |
| Clear cell carcinoma | 5 |
| Hodgkin lymphoma | 5 |
| Rhabdomyosarcoma | 4 |
| Sarcoma, NOS | 4 |
| Sebaceous carcinoma | 4 |
| Basal cell adenocarcinoma | 3 |
| Epithelial myoepithelial carcinoma | 3 |
| Oncocytic carcinoma | 3 |
| Adenosquamous carcinoma | 2 |
| Basal cell carcinoma** | 2 |
| Angiosarcoma | 1 |
| Burkitt lymphoma | 1 |
| Dermatofibrosarcoma protuberans** | 1 |
| Low-grade adenocarcinoma, NOS (primary salivary gland tumor) | 1 |
| Malignant lymphoma ex Warthin | 1 |
| Papillary cystadenocarcinoma | 1 |
| Plasmacytoma | 1 |
| Polymorphous (low-grade) adenocarcinoma | 1 |
| Polymorphous adenocarcinoma (coexisting with PA) | 1 |
| Undifferentiated pleomorphic sarcoma | 1 |
| Malignant peripheral nerve sheath tumor | 1 |
| Undifferentiated pleomorphic sarcoma, post-radiation origin | 1 |
| Carcinosarcoma | 1 |
| Metastases | 276 |
| Squamous cell carcinoma | 111 |
| Melanoma | 74 |
| Breast cancer | 18 |
| Retinoblastoma | 10 |
| Rhabdomyosarcoma | 8 |
| Basaloid squamous cell carcinoma | 8 |
| Sebaceous adenocarcinoma | 5 |
| Undifferentiated carcinoma of the nasopharynx | 5 |
| Chordoma | 3 |
| Merkel cell carcinoma | 3 |
| Neuroendocrine cancer | 3 |
| Small cell neuroendocrine carcinoma | 3 |
| Thyroid adenocarcinoma, NOS | 3 |
| Papillary thyroid carcinoma | 3 |
| Carcinoma, NOS | 2 |
| Colon cancer | 2 |
| Kidney cancer | 2 |
| Leiomyosarcoma | 2 |
| Lung cancer | 2 |
| Merkel cell cancer | 2 |
| Osteosarcoma | 2 |
| Appendix cancer | 1 |
| Astrocytoma | 1 |
| Chondrosarcoma | 1 |
| Sarcoma | 1 |
| Synovial sarcoma | 1 |

* The concept of “mucoepidermoid carcinoma ex pleomorphic adenoma” is not well accepted, as it is typically MAML2-driven and not derived from PLAG1- or HMGA2-positive pleomorphic adenomas.

** This case represents a cutaneous/soft tissue tumor involving the region overlying the parotid gland, not a primary or metastatic salivary gland tumor.
