# Supplementary material for: Comparative analysis of the World Health Organization Reporting System for Head and Neck Cytopathology and the Milan System for Reporting Salivary Gland Cytopathology
Source: Cancer Cytopathol. 2025 Aug 25;133(9):e70041. doi: 10.1002/cncy.70041 (PMC12377497; doi:10.1002/cncy.70041)
Supplement: Supplementary file 2 — Table S2 [file CNCY-133-0-s001.docx]

**Supplementary Table 2.** False negative FNA diagnoses with corresponding histological findings

| No. | Milan class | Age | FNA diagnosis | Histological diagnosis |
| --- | --- | --- | --- | --- |
| 1. | I. Nondiagnostic | 61 | Inflammation | Acinic cell carcinoma |
| 2. | II. Non-neoplastic | 59 | Lymph node | Melanoma |
| 3. | II. Non-neoplastic | 43 | Lymph node | Mucoepidermoid carcinoma |
| 4. | II. Nonneoplastic | 57 | Inflammation | Mucoepidermoid carcinoma ex pleomorphic adenoma |
| 5. | II. Non-neoplastic | 54 | Inflammation | Mucoepidermoid carcinoma |
| 6. | II. Non-neoplastic | 65 | Inflammation | Mucoepidermoid carcinoma |
| 7. | II. Non-neoplastic | 68 | Inflammation | Squamous cell carcinoma |
| 8. | II. Non-neoplastic | 79 | Inflammation | Squamous cell carcinoma |
| 9. | II. Nonneoplastic | 69 | Inflammation | Squamous cell carcinoma |
| 10. | II. Nonneoplastic | 81 | Inflammation | Squamous cell carcinoma |
| 11. | II. Nonneoplastic | 59 | Inflammation | Leiomyosarcoma |
| 12. | II. Nonneoplastic | 63 | Adenitis | Non-Hodgkin lymphoma |
| 13. | II. Nonneoplastic | 48 | Adenitis | Non-Hodgkin lymphoma |
| 14. | II. Nonneoplastic | 47 | Lymphoid hyperplasia | Non-Hodgkin lymphoma |
| 15. | II. Nonneoplastic | 72 | Lymphoid hyperplasia | Malignant lymphoma ex Warthin |
| 16. | II. Nonneoplastic | 55 | Lymphoid hyperplasia | Mucoepidermoid carcinoma |
| 17. | II. Nonneoplastic | 65 | Epidermal cyst | Mucoepidermoid carcinoma |
| 18. | II. Nonneoplastic | 45 | Epidermal cyst | Mucoepidermoid carcinoma |
| 19. | II. Nonneoplastic | 46 | Epidermal cyst | Mucoepidermoid carcinoma |
| 20. | II. Nonneoplastic | 9 | Epidermal cyst | Mucoepidermoid carcinoma |
| 21. | II. Nonneoplastic | 9 | Epidermal cyst | Mucoepidermoid carcinoma |
| 22. | II. Nonneoplastic | 60 | Epidermal cyst | Papillary carcinoma |
| 23. | II. Nonneoplastic | 73 | Normal cells | Mucoepidermoid carcinoma |
| 24. | II. Nonneoplastic | 39 | Normal cells | Mucoepidermoid carcinoma |
| 25. | II. Nonneoplastic | 63 | Normal cells | Adenoid cystic carcinoma |
| 26. | II. Nonneoplastic | 57 | Normal cells | Osteosarcoma |
| 27. | IVa. Benign neoplasm | 33 | Pleomorphic adenoma | Low-grade adenocarcinoma, NOS (primary salivary gland tumor) |
| 28. | IVa. Benign neoplasm | 80 | Pleomorphic adenoma | Low-grade adenocarcinoma, NOS (primary salivary gland tumor) |
| 29. | IVa. Benign neoplasm | 50 | Pleomorphic adenoma | Squamous cell carcinoma ex pleomorphic adenoma |
| 30. | IVa. Benign neoplasm | 72 | Pleomorphic adenoma | Basaloid squamous cell carcinoma |
| 31. | IVa. Benign neoplasm | 61 | Pleomorphic adenoma | Adenoid cystic carcinoma |
| 32. | IVa. Benign neoplasm | 70 | Pleomorphic adenoma | Adenoid cystic carcinoma |
| 33. | IVa. Benign neoplasm | 70 | Pleomorphic adenoma | Acinic cell carcinoma |
| 34. | IVa. Benign neoplasm | 38 | Pleomorphic adenoma | Acinic cell carcinoma |
| 35. | IVa. Benign neoplasm | 56 | Pleomorphic adenoma | Carcinoma ex pleomorphic adenoma |
| 36. | IVa. Benign neoplasm | 62 | Pleomorphic adenoma | Carcinoma ex pleomorphic adenoma, not otherwise specified |
| 37. | IVa. Benign neoplasm | 56 | Pleomorphic adenoma | Carcinoma ex pleomorphic adenoma, not otherwise specified |
| 38. | IVa. Benign neoplasm | 29 | Pleomorphic adenoma | Carcinoma ex pleomorphic adenoma, not otherwise specified |
| 39. | IVa. Benign neoplasm | 71 | Pleomorphic adenoma | Carcinoma, not otherwise specified |
| 40. | IVa. Benign neoplasm | 15 | Pleomorphic adenoma | Myoepithelial carcinoma ex pleomorphic adenoma |
| 41. | IVa. Benign neoplasm | 66 | Pleomorphic adenoma | Myoepithelial carcinoma ex pleomorphic adenoma |
| 42. | IVa. Benign neoplasm | 77 | Pleomorphic adenoma | Low-grade papillary adenocarcinoma ex pleomorphic adenoma |
| 43. | IVa. Benign neoplasm | 36 | Pleomorphic adenoma | Clear cell carcinoma ex pleomorphic adenoma |
| 44. | IVa. Benign neoplasm | 38 | Pleomorphic adenoma | Mucoepidermoid carcinoma ex pleomorphic adenoma |
| 45. | IVa. Benign neoplasm | 45 | Oncocytoma | Mucoepidermoid carcinoma |
| 46. | IVa. Benign neoplasm | 17 | Basal cell adenoma | Mucoepidermoid carcinoma |
| 47. | IVa. Benign neoplasm | 42 | Vascular tumor | Carcinoma, not otherwise specified |
| 48. | IVa. Benign neoplasm | 56 | Warthin's tumor | Mucoepidermoid carcinoma |
| 49. | IVb. Salivary gland neoplasm of uncertain malignant potential | 25 | Fusiform cells | Myoepithelial carcinoma |
