# Supplementary material for: Comparative analysis of the World Health Organization Reporting System for Head and Neck Cytopathology and the Milan System for Reporting Salivary Gland Cytopathology
Source: Cancer Cytopathol. 2025 Aug 25;133(9):e70041. doi: 10.1002/cncy.70041 (PMC12377497; doi:10.1002/cncy.70041)
Supplement: Supplementary file 3 — Table S3 [file CNCY-133-0-s002.docx]

**Supplementary Table 3.** False positive FNA diagnoses with corresponding histological findings

| **No.** | **Milan class** | **Age** | **FNA diagnosis** | **Histological diagnosis** |
| --- | --- | --- | --- | --- |
| 1. | V. Suspicious for malignancy | 41 | Pleomorphic adenoma vs. Adenoid cystic carcinoma | Pleomorphic adenoma |
| 2. | V. Suspicious for malignancy | 42 | Pleomorphic adenoma vs. Adenoid cystic carcinoma | Pleomorphic adenoma |
| 3. | V. Suspicious for malignancy | 41 | Pleomorphic adenoma vs. Adenoid cystic carcinoma | Pleomorphic adenoma |
| 4. | V. Suspicious for malignancy | 57 | Pleomorphic adenoma vs. Adenoid cystic carcinoma | Myoepithelioma |
| 5. | V. Suspicious for malignancy | 57 | Pleomorphic adenoma vs. Adenoid cystic carcinoma | Pleomorphic adenoma |
| 6. | V. Suspicious for malignancy | 30 | Pleomorphic adenoma suspicious for malignancy | Pleomorphic adenoma |
| 7. | V. Suspicious for malignancy | 59 | Pleomorphic adenoma suspicious for malignancy | Pleomorphic adenoma |
| 8. | V. Suspicious for malignancy | 52 | Pleomorphic adenoma suspicious for malignancy | Pleomorphic adenoma |
| 9. | V. Suspicious for malignancy | 53 | Pleomorphic adenoma suspicious for malignancy | Pleomorphic adenoma |
| 10. | V. Suspicious for malignancy | 74 | Pleomorphic adenoma suspicious for malignancy | Pleomorphic adenoma |
| 11. | V. Suspicious for malignancy | 90 | Pleomorphic adenoma suspicious for malignancy | Pleomorphic adenoma |
| 12. | V. Suspicious for malignancy | 58 | Suspicion of squamous cell carcinoma | Inflammation |
| 13. | V. Suspicious for malignancy | 30 | Suspicion of adenoid cystic carcinoma | Pleomorphic adenoma |
| 14. | V. Suspicious for malignancy | 50 | Suspicion of mucoepidermoid carcinoma | Epidermal cyst |
| 15. | V. Suspicious for malignancy | 24 | Suspicion of mucoepidermoid carcinoma | Pleomorphic adenoma |
| 16. | V. Suspicious for malignancy | 1 | Suspicion of fusiform cells sarcoma | Fibromatosis |
| 17. | V. Suspicious for malignancy | 4 | Atypical tumor of conjunctiva | Nodular fasciitis |
| 18. | V. Suspicious for malignancy | 46 | Warthin's tumor vs. Mucoepidermoid carcinoma | Warthin's tumor |
| 19. | V. Suspicious for malignancy | 36 | Necrosis | Tuberculosis |
| 20. | V. Suspicious for malignancy | 70 | Necrosis | Warthin's tumor |
| 21. | V. Suspicious for malignancy | 56 | Necrosis, Suspicious for malignancy | Benign lymphoepithelial lesion |
| 22. | V. Suspicious for malignancy | 64 | Suspicious for malignancy | Adamantinoma |
| 23. | V. Suspicious for malignancy | 73 | Suspicious for malignancy | Tuberculosis |
| 24. | V. Suspicious for malignancy | 28 | Suspicious for malignancy | Pleomorphic adenoma |
| 25. | V. Suspicious for malignancy | 51 | Suspicious for malignancy | Schwannoma |
| 26. | V. Suspicious for malignancy | 63 | Suspicious for malignancy | Basal cell adenoma |
| 27. | V. Suspicious for malignancy | 10 | Suspicious for malignancy | Pilomatricoma |
| 28. | V. Suspicious for malignancy | 84 | Suspicious for malignancy | Inflammation |
| 29. | V. Suspicious for malignancy | 38 | Suspicious for malignancy | Mucocele |
| 30. | VI. Malignant | 83 | Urothelial carcinoma | Inverted ductal papilloma |
| 31. | VI. Malignant | 81 | Squamous cell carcinoma | Warthin’s tumor |
| 32. | VI. Malignant | 63 | Squamous cell carcinoma | IgG4-related disease |
| 33. | VI. Malignant | 60 | Mucoepidermoid carcinoma | IgG4-related disease |
| 34. | VI. Malignant | 57 | Mucoepidermoid carcinoma | Basal cell adenoma |
| 35. | VI. Malignant | 39 | Adenoid cystic carcinoma | Pleomorphic adenoma |
| 36. | VI. Malignant | 79 | Adenoid cystic carcinoma | Pleomorphic adenoma |
| 37. | VI. Malignant | 68 | Acinic cell carcinoma | Pleomorphic adenoma |
| 38. | VI. Malignant | 63 | Adenocarcinoma | Pleomorphic adenoma |
| 39. | VI. Malignant | 54 | Carcinoma | Pleomorphic adenoma |
| 40. | VI. Malignant | 22 | Spindle cell tumor | Nodular fasciitis |
